# Enhanced Heavy Metal Tolerance and Accumulation by Transgenic Sugar Beets Expressing *Streptococcus* thermophilus StGCS-GS in the Presence of Cd, Zn and Cu Alone or in Combination

**DOI:** 10.1371/journal.pone.0128824

**Published:** 2015-06-09

**Authors:** Dali Liu, Zhigang An, Zijun Mao, Longbiao Ma, Zhenqiang Lu

**Affiliations:** 1 Key Laboratory of Sugar beet Genetics and Breeding, Academy of Crop Sciences, Heilongjiang University, Harbin, 150080, P. R. China; 2 Key Laboratory of Forest Plant Ecology, the Ministry of Education of China, Northeast Forestry University, Harbin, 150040, P. R. China; 3 Key Laboratory of Biochemistry and Molecular Biology, College of Life Sciences, Heilongjiang University, Harbin, 150080, P. R. China; National Taiwan University, TAIWAN

## Abstract

Phytoremediation is a promising means of ameliorating heavy metal pollution through the use of transgenic plants as artificial hyperaccumulators. A novel *Streptococcus thermophilus* *γ*-glutamylcysteine synthetase-glutathione synthetase (StGCS-GS) that synthesizes glutathione (GSH) with limited feedback inhibition was overexpressed in sugar beet (*Beta vulgaris* L.), yielding three transgenic lines (s2, s4 and s5) with enhanced tolerance to different concentrations of cadmium, zinc and copper, as indicated by their increased biomass, root length and relative growth compared with wild-type plants. Transgenic sugar beets accumulated more Cd, Zn and Cu ions in shoots than wild-type, as well as higher GSH and phytochelatin (PC) levels under different heavy metal stresses. This enhanced heavy metal tolerance and increased accumulation were likely due to the increased expression of StGCS-GS and consequent overproduction of both GSH and PC. Furthermore, when multiple heavy metal ions were present at the same time, transgenic sugar beets overexpressing StGCS-GS resisted two or three of the metal combinations (50 μM Cd-Zn, Cd-Cu, Zn-Cu and Cd-Zn-Cu), with greater absorption in shoots. Additionally, there was no obvious competition between metals. Overall, the results demonstrate the explicit role of StGCS-GS in enhancing Cd, Zn and Cu tolerance and accumulation in transgenic sugar beet, which may represent a highly promising new tool for phytoremediation.

## Introduction

Cadmium, zinc and copper ions are toxic at certain concentrations and can pose threats to plants and the environment. Additionally, these ions are hazardous to human health when they accumulate in the food chain. Phytoremediation is a highly efficient, economical and environmentally friendly means of removing heavy metals from contaminated soil. During the remediation process, these toxic elements are extracted or stabilized by plants and metabolized in their tissues [1]. Naturally existing “hyperaccumulators” tend to be small and have low biomass production. Increased heavy metal tolerance and accumulation capacity could be conferred in plants by transferring functional genes via genetic engineering. Sugar beet (*Beta vulgaris* L.) is an emerging renewable energy crop with a high biomass and ethanol conversion rate and a strong ability to adapt to the environment, making it a suitable target species for phytoremediation.

In plant cells, glutathione (GSH) is a major redox buffer for ionic homeostasis and detoxification in heavy metal defense mechanisms [2]. GSH acts as an antioxidant in the defense against reactive oxygen species (ROS) through the ascorbate-glutathione cycle upon heavy metal exposure [3]. In addition, GSH is a major reservoir of non-protein reduced sulfur for the chelation of heavy metal ions [4]. GSH is also the direct precursor of phytochelatins (PCs), which are the principal class of heavy metal chelators that facilitate metal sequestration in vacuoles [5]. These characteristics, along with the relative stability and high water solubility of GSH, make it an ideal molecule for the protection of plants against heavy metal stresses [6].

GSH synthesis is catalyzed by *γ*-glutamylcysteine synthetase (*γ*-GCS, GSH1; EC 6.3.2.2) and glutathione synthetase (GS, GSH2; EC 6.3.2.3) in eukaryotic cells and nearly all Gram-negative bacteria, and *γ*-GCS is feedback inhibited by GSH [7]. Several genes participating in GSH production have been identified and transferred into different organisms to confer improved tolerance to specific heavy metal stress conditions. Transgenic plants overexpressing the *Escherichia coli GSH1* and *GSH2* genes accumulated significantly more Cd ions, exhibited enhanced tolerance to Cd and accumulated higher concentrations of GSH, PC and thiols than wild-type (wt) [8, 9]. Similarly, overexpression of a bacterial *GSH1* gene in the cytosol or chloroplast of *Populus canescens* increased GSH levels and conferred zinc tolerance to the transgenic plants [10]. Overexpression of *γ*-EC and PCS1 in transgenic tobacco resulted in increased Cd tolerance via the accumulation of ions and elevated thiol levels [11]. When *Agrostis palustris* was transformed with *Phragmites australis PaGCS*, the transgenic plants exhibited more effective growth than wt under Cd stress due to increased accumulation of Cd^2+^ and PC [12]. Overexpressing *GSH1* and *AsPCS1* also increased the tolerance to and accumulation of cadmium and arsenic in *Arabidopsis thaliana*, while at the same time, the GSH and PC contents increased [13]. These findings suggest that the increased demand for GSH is an essential component of plant tolerance to and accumulation of various heavy metals, and they provide support for the development of transgenic plants overexpressing genes involved in GSH synthesis, which exhibit increased heavy metal resistance and uptake, for phytoremediation purposes.

A novel enzyme involved in GSH synthesis, *γ*-glutamylcysteine synthetase-glutathione synthetase (StGCS-GS), was recently identified in *Streptococcus thermophiles*. StGCS-GS was neither redox-regulated nor sensitive to feedback inhibition by GSH [14]. *E*. *coli* overexpressing StGCS-GS showed significant enhancement of Cd, Zn and Cu tolerance with considerable increases in heavy metal accumulation and GSH content compared with *E*. *coli* overexpressing *Arabidopsis γ*-glutamylcysteine synthetase (AtGCS) and glutathione synthetase (AtGS) [15]. We therefore sought to explore the application of the heavy metal tolerance of StGCS-GS in phytoremediation. Thus, in the present study, sugar beet was transformed with StGCS-GS to generate three stably expressing transgenic lines, which were then subjected to specific concentrations of Cd, Zn and Cu individually or simultaneously. Heavy metal tolerance and accumulation were examined comprehensively using both GSH and PC content assays. The results obtained clearly indicated an essential role for StGCS-GS in the bioremediation of single and multiple toxic metal ions in transgenic sugar beet.

## Materials and Methods

### Plant materials and growth conditions

Sugar beet (*Beta vulgaris* L.) seeds (US-8916) were surface-sterilized with 0.1% (w/v) mercuric chloride for 8 min, followed by thorough rinses with sterile water. They were then sown on plates containing 1/2 Murashige and Skoog (MS) medium with 3% sucrose [16]. After germination for 4 to 7 days, the seedlings were transferred to solid MS medium supplemented with 0.5 mg L^-1^ 6-BA and 3% sucrose for bud differentiation. MS medium containing 1.5 mg L^-1^ NAA was used for growing and rooting. The plants were maintained in a culture room at 23°C under a 16-h photoperiod.

### Construction of a plant expression cassette and transformation of sugar beet

The coding region of StGCS-GS (GenBank accession no. GQ848551) [14] was amplified using the primers 5′-**CACC**ATGACATTAAACCAACTGCTT-3′ and 5′- TTAAGTTTGACCAGCCACTATTTCT-3′ and *Pfu* DNA polymerase (*Trans*Gen, CHN). The PCR product was inserted into pENTR/SD/D-TOPO (Invitrogen) and recombined with the destination vector pGWB2 (GATEWAY system) to generate the expression clone pGWB2-StGCS-GS for transformation. This expression vector was introduced into the *Agrobacterium tumefaciens* strain EHA105 by electroporation. After 2 days of pre-cultivation on bud differentiation medium, multiple shoots of sugar beet were inoculated with a suspension of the StGCS-GS-containing EHA105 suspended in MS liquid medium for 15 min. Co-cultivation was continued for 4 days on bud differentiation medium. Then, the co-cultivated materials were transferred to selective medium (MS medium supplemented with 50 mg L^-1^ hygromycin (Roche) for selection of stable transformants and 300 mg L^-1^ cefotaxime to prevent growth of bacteria) for 1 month. Each putative transformant (hygromycin-resistant bud) was micropropagated to produce twenty clones for further examination.

### Semi-quantitative reverse transcription PCR analysis

The expression level of StGCS-GS in the hygromycin-resistant transformants was confirmed by semi-quantitative RT-PCR using the primers 5′-GCGTTGTGAGGCTGTTCT-3′ and 5′-AATGCAGGTGCAATGAGG-3′. *Beta vulgaris Actin 1* (*BvActin 1*, GenBank accession no. DQ866829) was used as a standard control and was amplified with the primers 5′-CCCACTGAATCCCAAGGC-3′ and 5′-TTTCCCGTTCGGCTGATG-3′. Total RNA was isolated independently from control (wt) and transgenic beets using Trizol Reagent (Invitrogen). cDNA was prepared using *Trans*Script First-Strand cDNA Synthesis SuperMix (*Trans*Gen). The amplified PCR products were analyzed on a 1.0% agarose gel, and the band intensity was quantified using Quantity One software.

### Assessment of the heavy metal tolerance of transgenic plants

To assess the relative heavy metal tolerance of various plants, one-week-old transgenic and wild-type sugar beet buds with a height of 1 cm and three leaves were grown on rooting medium (MS + 1.5 mg L^-1^ NAA) supplemented with 0, 50, 100 or 200 μM Cd^2+^ (CdCl_2_), Zn^2+^ (ZnCl_2_) or Cu^2+^ (CuCl_2_). For the complex heavy metal stress assays, combinations of two or three ions at 50 μM were added to the previously described medium. After 3 weeks of treatment, the shoots and roots of all tested plants were harvested and thoroughly rinsed with distilled water to remove adherent culture medium. The heavy metal tolerance of the plants was estimated by measuring the fresh weight (FW) and root length. For the hydroponic experiment, the one-week-old seedlings were transferred to pots containing one-half-strength Hoagland solution. After 3 weeks of growth, the nutrient solution was replaced with fresh solution amended with heavy metal ions at the same concentrations described above. Untreated plants were grown in parallel under the same conditions. Plants were harvested after 2 weeks of incubation, and total fresh weights were measured before and after the treatment to determine the effect of different heavy metal stresses on growth. All experiments were repeated three times.

### Heavy metal ion accumulation assay

Samples were dried at 105°C for 30 min and then transferred to 80°C until a constant weight was achieved; this was recorded as the dry weight (DW). These dry tissues were ground and digested in HCl at 100°C for 30 min. Digested samples were diluted with HNO_3_, HF and HClO_4_ at 170°C for 1 h. The Cd, Zn and Cu contents were determined using an atomic absorption spectrometer (Thermo Electron Corp., USA).

### Determination of GSH and PC contents

Fresh samples (100 mg) were frozen in liquid nitrogen and ground into powder. The tissue was then homogenized in 5% 5-sulfosalicylic acid (SSA). After centrifuging at 8000 × g for 10 min, the supernatant was transferred to a new tube for a total GSH assay using a Total Glutathione Quantification Kit (DoJinDo Molecular Technologies, Inc.). The GSH concentration is expressed as μmol g^-1^ fresh weight (FW).

For PC content determination, the above supernatant was mixed with K_2_HPO_4_ and 10 mM DNTB to analyze non-protein thiol (NPT) content at OD_412_. The PC concentration was determined as the difference between NPT and GSH and expressed as μmol g^-1^ fresh weight (FW).

### Statistical analysis

All experiments were performed in triplicate, and 3 seedlings of each examined line were analyzed in each treatment. The values shown in the tables and figures are the mean values ± standard deviation (SD). The means were compared by one-way analysis of variance and Student’s *t* test at a 5% level of significance.

## Results

### Development of StGCS-GS transgenic sugar beet plants

**Fig 1:**
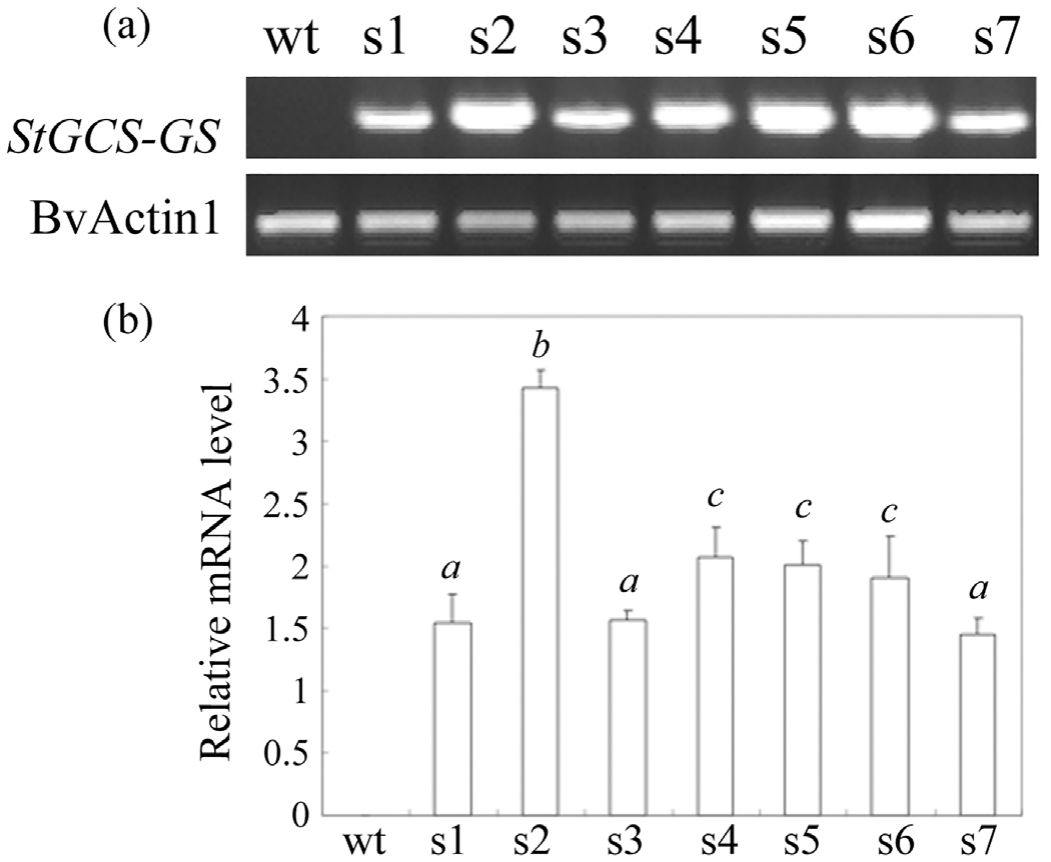
Detection of StGCS-GS transcripts by semi-quantitative RT-PCR in transgenic sugar beets. (a) Semi-quantitative RT-PCR analysis of seven transgenic sugar beet (*Beta vulgaris* L.) lines overexpressing StGCS-GS (s1 to s7). BvActin1 was used as a loading control. The PCR products were separated by electrophoresis on agarose gels and visualized by staining with ethidium bromide. (b) The intensity of each band was measured using Quantity One software. The blank bars represent the StGCS-GS/BvActin1 expression intensity ratios. Different italicized letters (*a-c*) indicate expression differences (*P*<0.05) between lines. wt: wild-type.

Transgenic sugar beet seedlings overexpressing StGCS-GS were generated by introducing the StGCS-GS cassette from pGWB2-StGCS-GS. Multiple shoots with a high differentiation capacity were transformed with StGCS-GS via *Agrobacterium*-mediated transformation. Seven independent hygromycin-resistant lines were obtained after transformation and designated s1 to s7. The transcript levels of StGCS-GS in the hygromycin-resistant lines were examined by semi-quantitative reverse transcription PCR (Fig 1). All transgenic lines yielded the StGCS-GS product when specific primers were used, whereas no product was obtained with the control plants (wild-type, wt), confirming that StGCS-GS was constitutively expressed in the transgenic sugar beets. Different lines exhibited different expression levels, and transgenic lines s2, s4 and s5, which exhibited relatively higher StGCS-GC expression levels, were selected for subsequent examination under cadmium, zinc and copper stress.

### Overexpression of StGCS-GS improves Cd, Zn and Cu tolerance in transgenic sugar beets

**Fig 2:**
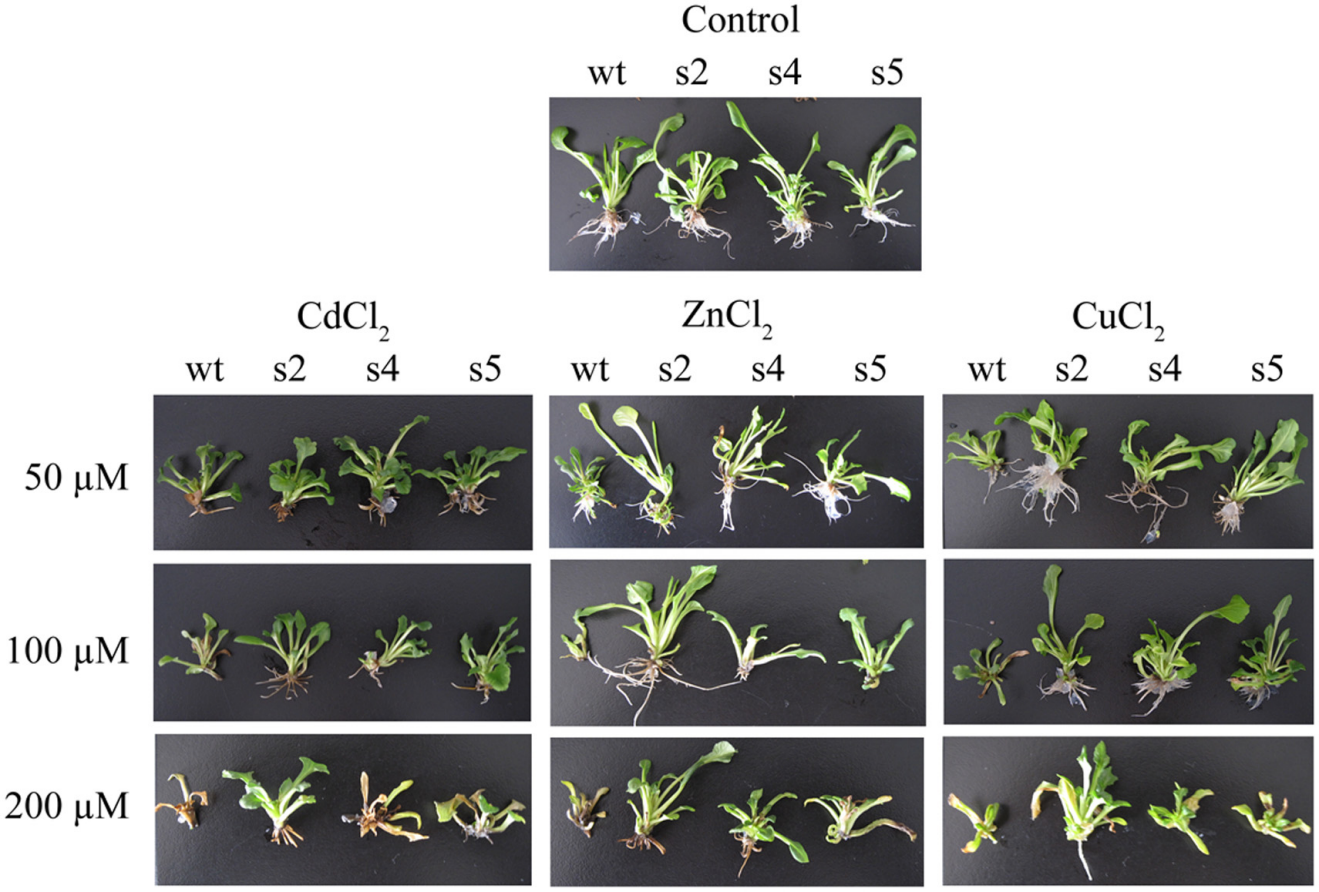
Enhancement of heavy metal tolerance by overexpression of StGCS-GS in transgenic sugar beets. One-week-old transgenic seedlings (s2, s4 and s5) and wild-type seedlings (wt) were grown for 3 weeks on MS medium +1.5 mg L^-1^ NAA supplemented with 0 (Control), 50, 100 or 200 μM CdCl_2_, ZnCl_2_ or CuCl_2_.

To determine whether overexpression of StGCS-GS in sugar beet enhances heavy metal tolerance, transgenic (s2, s4 and s5) and wt plants were treated with 0, 50, 100 or 200 μM Cd^2+^ (CdCl_2_), Zn^2+^ (ZnCl_2_) or Cu^2+^ (CuCl_2_) (Fig 2). No obvious differences among the four lines were observed under the control conditions. However, when the plants were treated with increasing concentrations of Cd, Zn or Cu, the wt sugar beets displayed progressive chlorosis, growth inhibition, and decreased vigor. Wt plants treated with any of the metals at 200 μM nearly died. In contrast, all three transgenic lines exhibited markedly enhanced heavy metal tolerance at 50 and 100 μM heavy metal (Fig 2). In general, s2 exhibited better growth performance than s4 and s5, and it displayed relatively high tolerance to 200 μM stress. The transgenic lines were more tolerant to Zn and Cu than Cd. When suffered suffering from the severe stresses of exposure to 300 μM heavy metal ions, the transgenic lines also exhibited obvious growth inhibition with the performance of almost no root elongation (data not shown).

**Fig 3:**
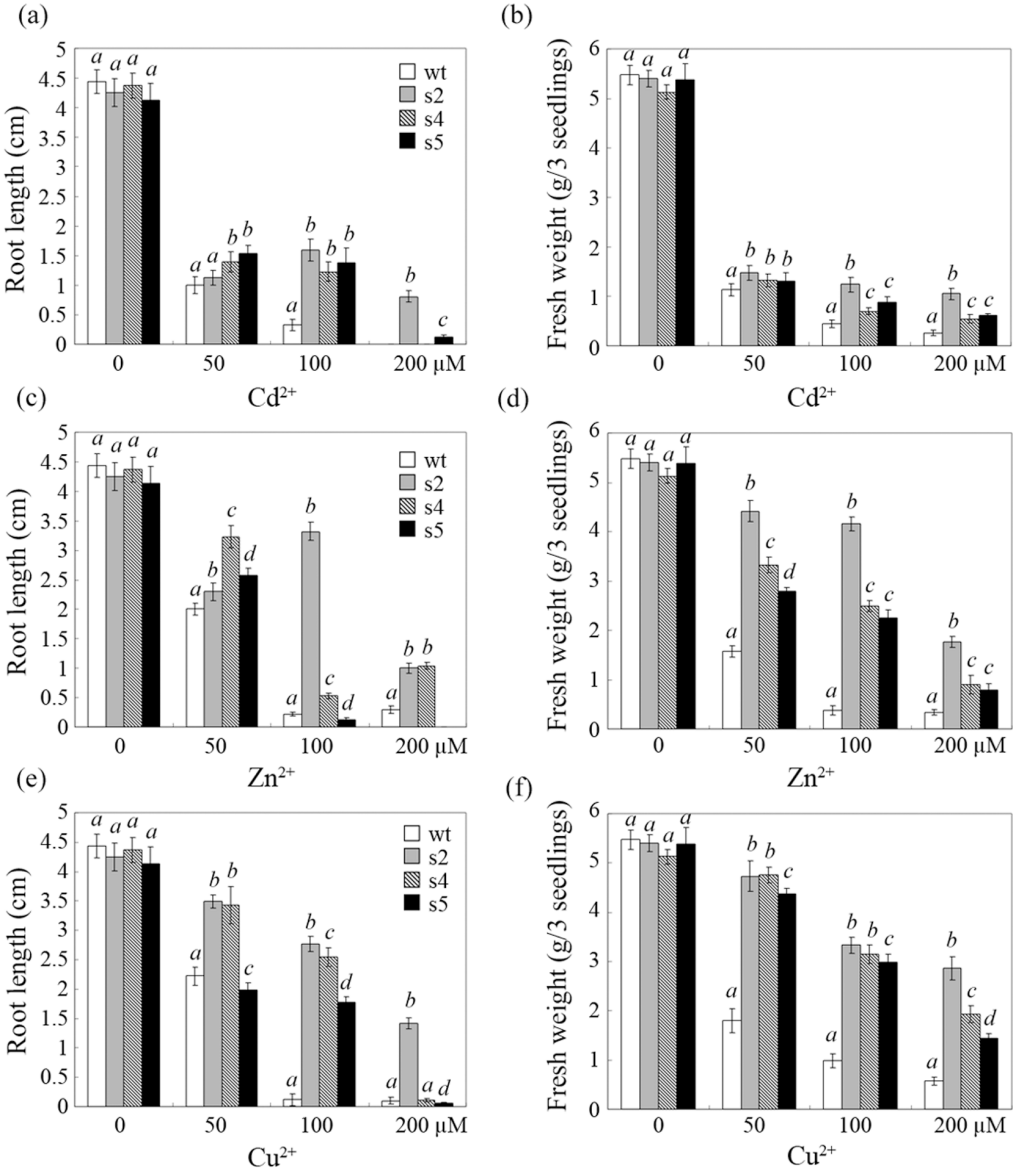
Comparative analysis of the root length and fresh weight of transgenic lines under heavy metal stress. Root lengths (a, c and e) and fresh weights (b, d and f) of transgenic sugar beets overexpressing StGCS-GS (s2, s4 and s5) and wild-type sugar beets (wt) were measured after 3 weeks of treatment with 0, 50, 100 or 200 μM Cd^2+^, Zn^2+^ or Cu^2+^. The experiments were performed in triplicate, and the values are presented as the means ± SD. Means denoted by the same italicized letter did not differ significantly (*P*<0.05).

We further analyzed the heavy metal tolerance of each transgenic line in detail by measuring root length and fresh weight (Fig 3). In contrast to the similarities in biomass and root growth observed among wt and transgenic plants in the absence of heavy metal stress, the different ions affected the four tested lines differently. Cd reduced the root length and fresh weight of all tested lines more severely than Zn and Cu. The wt root length was only approximately 1/5 of that of s2 in the presence of 100 μM Cd, and root elongation was nearly undetectable at 200 μM Cd (Fig 3a). The fresh weights of s2, s4 and s5 were approximately 2- to 3-fold greater than that of wt at 100 and 200 μM Cd (Fig 3b). Under different concentrations of Zn and Cu, transgenic plants consistently exhibited significantly enhanced root length and fresh weight compared to wt. In particular, at 100 μM Zn or Cu, the root length of s2 was approximately 16-fold greater than that of wt, and the fresh weights of s2, s4 and s5 were 6- to 9-fold greater than that of wt. The biomass of the transgenic lines was generally 2-fold greater than that of the wt plants under Cu treatment (Fig 3c–3f). Despite the improved characteristics of the transgenic lines, root elongation was not always correlated with the expression level of StGCS-GS, particularly at 50 μM Cd or Zn (Fig 3a and 3c).

**Fig 4:**
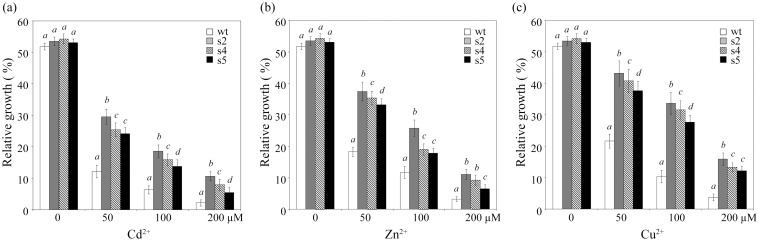
Growth inhibition of transgenic lines by heavy metal ions under hydroponic conditions. Both transgenic (s1, s2 and s3) and wild-type (wt) sugar beets were cultivated in one-half-strength Hoagland solution for 3 weeks and treated with 0, 50, 100 or 200 μM Cd (a), Zn (b) and Cu (c) for 14 days. Relative growth was expressed as the percentage increase in fresh weight of the same line during this period. The values shown are the average ± standard deviation. Significant differences (*P*<0.05) are indicated as italicized letters (*a-d*).

Similar results were also detected in hydroponic experiments. After two weeks of growth in nutrient solutions containing 0, 50, 100 and 200 μM CdCl_2_, ZnCl_2_ and CuCl_2_, the three transgenic lines showed superior heavy metal tolerance compared with the wt, as indicated by decreased growth inhibition (Fig 4). For example, the relative growth of s2 was approximately 40% in the presence of 100 μM Zn and Cu, whereas the relative growth of wt was only approximately 12%. The growth of s4 and s5 was intermediate between s2 and wt; this result is in accordance with the StGCS-GS expression levels (Figs 1 and 4). These results indicate that overexpression of StGCS-GS largely improved sugar beet heavy metal tolerance.

### Transgenic lines with superior heavy metal accumulation have increased GSH and PC contents

**Fig 5:**
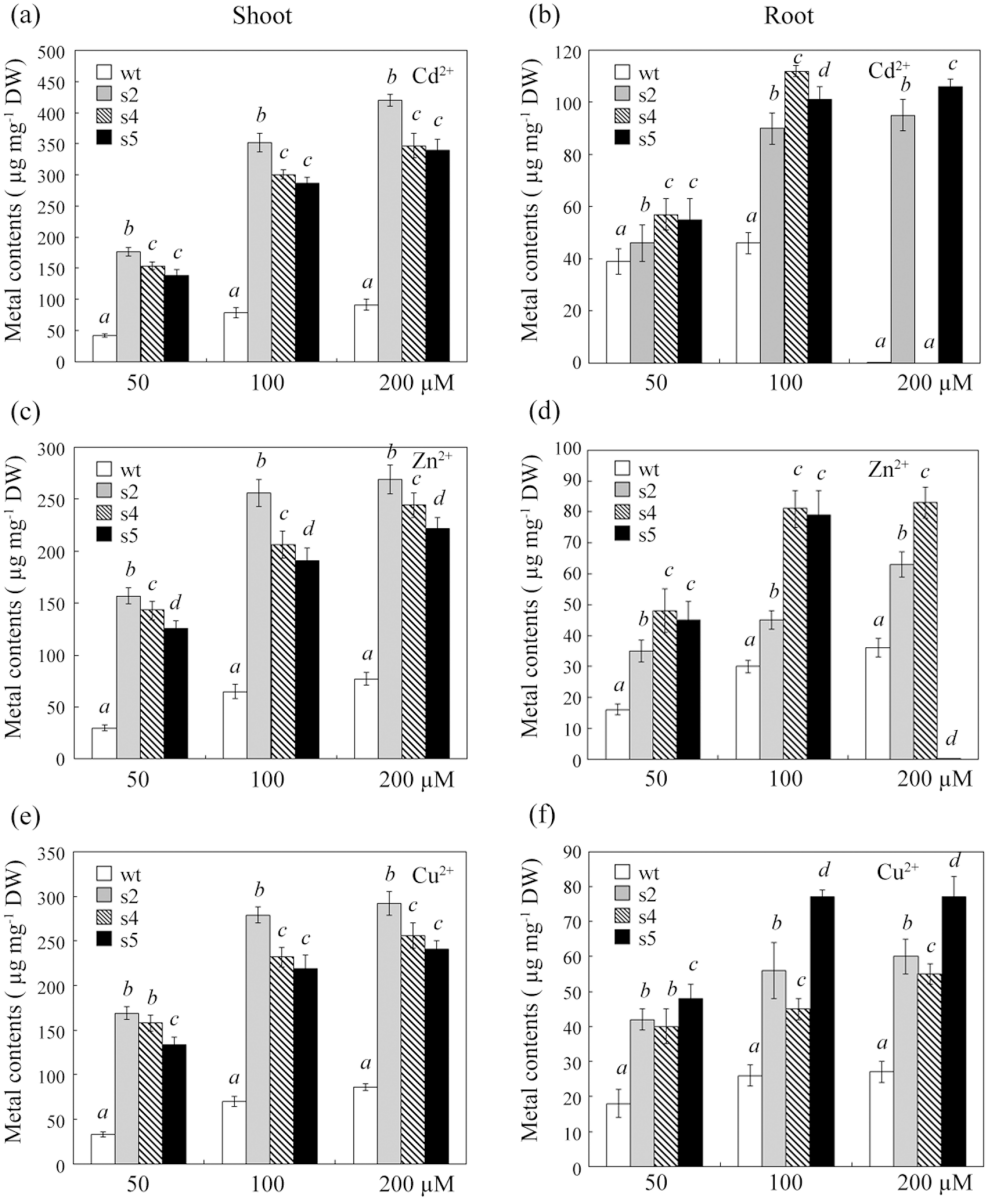
Heavy metal ion accumulation of transgenic sugar beets under Cd, Zn and Cu stress. The cadmium, zinc and copper content of shoots (a, c and e) and roots (b, d and f) of different transgenic lines was determined after exposure to 50, 100 or 200 μM CdCl_2_, ZnCl_2_ or CuCl_2_ for 3 weeks. wt: wild-type; s2, s4 and s5: different transgenic lines overexpressing StGCS-GS. The values shown are the means ± standard deviation. Different italicized letters (*a-d*) indicate significant differences (*P*<0.05) between lines.

The ability of the wt and the three transgenic sugar beet lines to expel or accumulate heavy metals was tested under 50, 100 or 200 μM Cd^2+^, Zn^2+^ or Cu^2+^. The quantities of ions in all tested lines increased as the external heavy metal concentration increased from 50 to 200 μM (Fig 5). Under pressure induced by 200 μM heavy metal concentrations, Cd content was not detected in wt and s4 specimens, and Zn content was not detected in s5 specimens; this phenomenon occurred because these specimens exhibited severely inhibited root growth. As expected, heavy metal concentrations in shoots and roots were significantly higher for all transgenic seedlings than for wt seedlings. Especially, nearly 4- to 5-fold increases in heavy metal accumulation were observed in shoots of s2, s4 and s5 compared to wt, following the trend s2>s4>s5>wt under 100 μM (Fig 5a, 5c and 5e). In roots, however, less Cd and Zn accumulated in s2 compared to s4 and s5, and the amount of Cu in s2 was lower than that in s5 but higher than that in s4. However, these ions were all present in higher amounts in the three transgenic lines compared with wt (Fig 5b, 5d and 5f). Overall, heavy metal accumulation was approximately three-fold higher in the aboveground parts compared with the belowground parts of transgenic plants.

**Fig 6:**
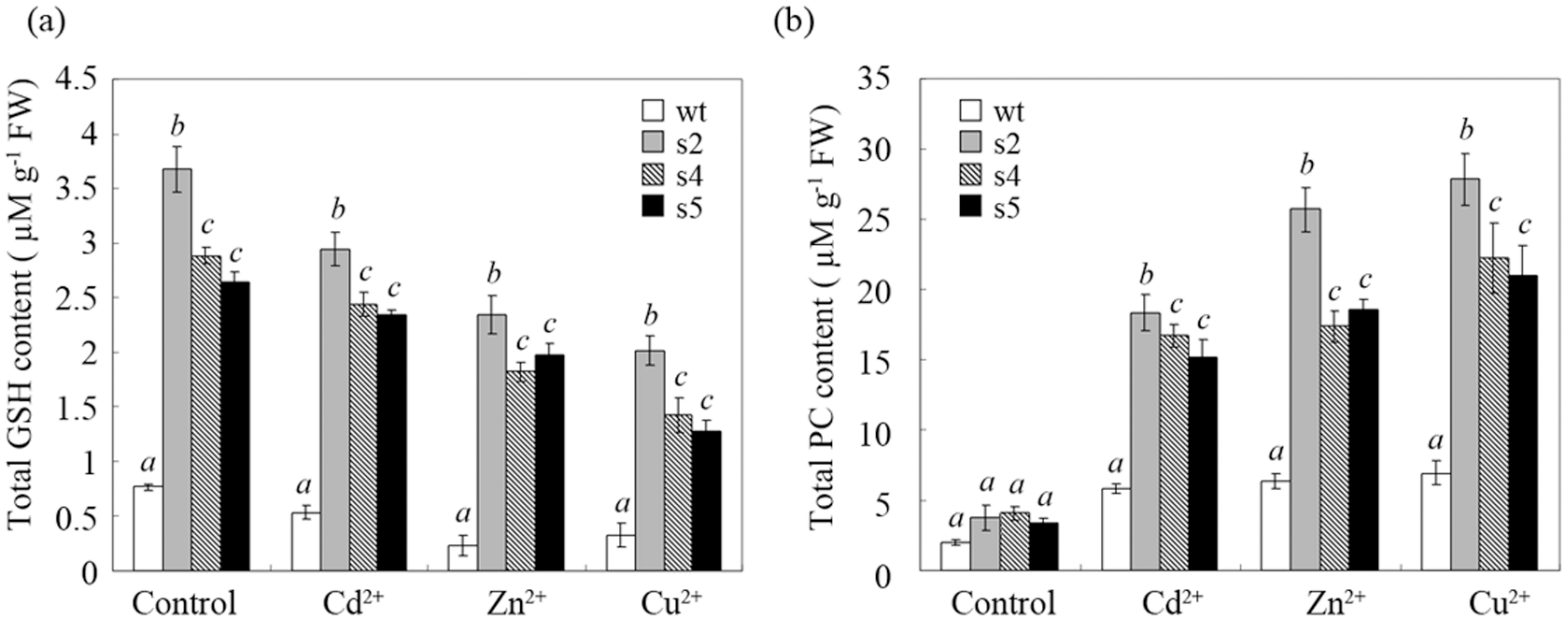
Glutathione and phytochelator content in different transgenic lines exposed to cadmium, zinc and copper. The total glutathione (GSH) (a) and phytochelator (PC) (b) contents of the transgenic lines were determined before (control) and after exposure to 100 μM CdCl_2_, ZnCl_2_ or CuCl_2_ for 3 weeks. wt: wild-type; s2, s4 and s5: transgenic lines overexpressing StGCS-GS. The values shown are means ± standard deviation. Different italicized letters (*a-c*) at the top of the error bars indicate significant differences (*P*<0.05).

Next, to investigate the effect of StGCS-GS overexpression on the production of heavy metal-binding compounds, the glutathione (GSH) and phytochelatin (PC) contents were measured under normal and 100 μM Cd, Zn or Cu stress conditions. As shown in Fig 6, under control conditions, the GSH contents of s2, s4 and s5 were 5-, 4- and 3.8-fold higher than that of wt, respectively, and there were no significant increases in PC content in the transgenic lines compared to wt. However, when exposed to 100 μM Cd^2+^, Zn^2+^ or Cu^2+^, the GSH content decreased in all tested lines, whereas the PC content increased, particularly under Cu stress. s2 exhibited the highest GSH and PC contents among all the tested lines, regardless of the stress condition. These data suggest that both GSH and PC contribute to heavy metal tolerance and accumulation.

### Transgenic sugar beets exhibit increased multiple heavy metal tolerance without selectivity among Cd, Zn and Cu

**Fig 7:**
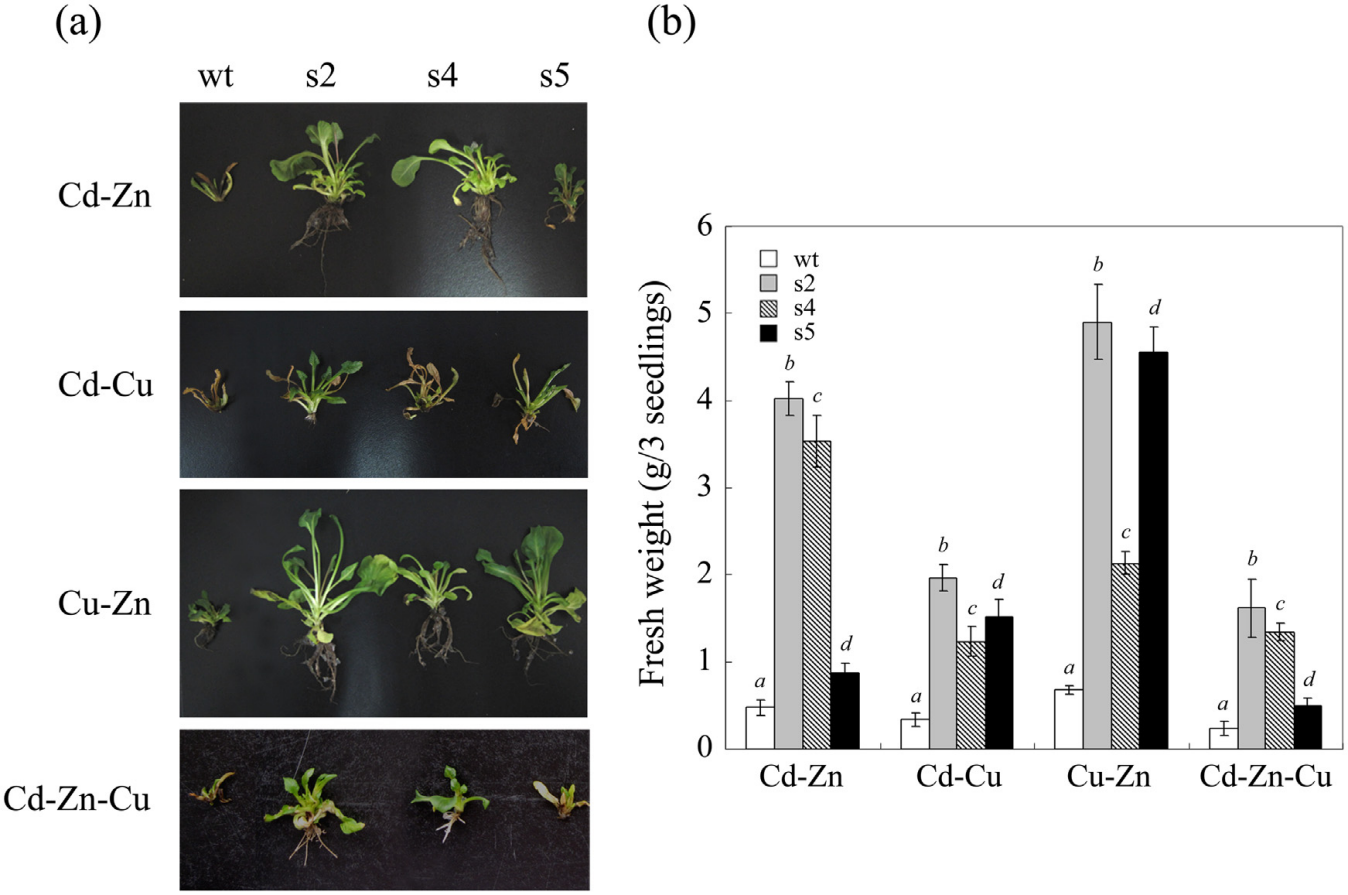
Multiple heavy metal tolerance of transgenic sugar beets overexpressing StGCS-GS. (a) One-week-old transgenic sugar beet lines overexpressing StGCS-GS (s2, s4 and s5) were grown on rooting medium (MS + 1.5 mg L^-1^ NAA) supplemented with combinations of two or three ions of 50 μM Cd, Zn or Cu (Cd-Zn, Cd-Cu, Zn-Cu or Cd-Zn-Cu) for 3 weeks. The wild-type seedlings (wt) were used as controls. (b) The fresh weight of all tested lines was measured three times, and the results are presented as the means ± SD. Different italicized letters indicate significant differences (*P*<0.05).

To evaluate the influence of multiple heavy metals on the transgenic sugar beet lines, all lines were treated with 50 μM Cd-Zn, Cd-Cu, Zn-Cu or Cd-Zn-Cu, and their heavy metal tolerance and ion absorption capacity were evaluated. As shown in Fig 7a, in contrast to the results observed upon treatment with single heavy metals at 50 μM, the growth of the wt plants was severely inhibited by treatment with 50 μM metal combinations (Cd-Zn, Cd-Cu, Zn-Cu and Cd-Zn-Cu), as indicated by yellow leaves, wilting, and a nearly complete lack of growth and rooting. However, overexpression of StGCS-GS improved the heavy metal tolerance of transgenic sugar beets under double and triple metal stress conditions. Cd-Zn-Cu and Cd-Cu treatment damaged plants more severely than Cd-Zn or Cu-Zn treatment. Meanwhile, fresh weight analysis revealed that the transgenic plants exhibited greater biomass under complex heavy metal stresses (Fig 7b). S2 continued to exhibit increased growth and biomass compared to the other two transgenic lines in these toxic environments.

Heavy metal ion accumulation was also determined under these complex heavy metal stress conditions. As shown in Table 1, in shoots, heavy metal accumulation followed the trend s2>s4>s5>wt, and the amounts of Cd, Zn and Cu in s2 transgenic beets were approximately 5-fold greater than those in wt on average. In roots, heavy metal accumulation could not be detected because the wt seedlings showed virtually no root growth or elongation, except in the Zn-Cu treatment at the lowest concentration. A similar effect was observed in s5 under Cd-Zn-Cu stress (Table 2). The three transgenic lines all displayed an increase in ion accumulation compared with wt, and s2 still exhibited the highest accumulation capacity among the transgenic lines and the highest expression level of StGCS-GS (Tables 1 and 2). Cd, Zn and Cu accumulation in the transgenic lines was relatively lower in the double or triple heavy metal treatments compared with the single heavy metal treatments, and accumulation remained concentrated in the shoots (Table 1). However, there was no obvious competitive or antagonistic relationship among Cd, Zn and Cu with respect to their accumulation in the transgenic sugar beets (Tables 1 and 2). These results further confirm that StGCS-GS-overexpressing transgenic sugar beets can tolerate complex heavy metal stresses via enhanced absorption of different ions, with no preference among metals.

**Table 1:** Accumulation of Cd, Zn and Cu ions in transgenic sugar beet shoots under complex heavy metal stress.

		Amount of heavy metal accumulated by transgenic sugar beet shoots [μg mg^-1^ dry weight (DW)]^a^			
Transgenic lines	Ions	Cd-Zn	Cd-Cu	Zn-Cu	Cd-Zn-Cu
wt	Cd^2+^	25.2±2.7a	21.9±1.5a	ND	17.6±1.1a
	Zn^2+^	18.7±1.6a	ND	23.2±1.6a	13.3±0.9a
	Cu^2+^	ND	20.2±1.3a	25.9±2.0a	15.6±1.3a
s2	Cd^2+^	135.6±9.6b	114.2±7.1b	ND	96.5±5.7b
	Zn^2+^	100.5±5.9b	ND	133.2±7.7b	78.7±4.9b
	Cu^2+^	ND	93.1±5.7b	139.8±8.9b	80.2±4.8b
s4	Cd^2+^	100.2±6.3c	56.6±5.4c	ND	73.2±5.2c
	Zn^2+^	66.3±4.2c	ND	78.9±5.2c	53.8±4.3c
	Cu^2+^	ND	52.5±3.9c	80.3±5.3c	60.5±4.4c
s5	Cd^2+^	56.8±4.6d	69.8±5.1d	ND	22.2±2.1d
	Zn^2+^	33.9±3.3d	ND	100.1±6.1d	16.4±1.5a
	Cu^2+^	ND	41.6±3.2d	106.9±5.8d	16.5±1.1a

^a^ Transgenic sugar beet lines overexpressing StGCS-GS (s2, s4 and s5) and wild-type sugar beets (wt) were treated with double or triple combinations of 50 μM CdCl_2_, ZnCl_2_ and CuCl_2_, designated as Cd-Zn, Cd-Cu, Zn-Cu and Cd-Zn-Cu. Shoots were collected for analysis of ion accumulation in triplicate. Differences in the means ± standard deviation with a 5% level of significance are indicated (*a-d*). ND: not detected.

**Table 2:** Accumulation of Cd, Zn and Cu ions in the roots of transgenic sugar beets under complex heavy metal stress.

		Amount of heavy metal accumulated by transgenic sugar beet roots [μg mg^-1^ dry weight (DW)]^a^			
Transgenic lines	Ions	Cd-Zn	Cd-Cu	Zn-Cu	Cd-Zn-Cu
wt	Cd^2+^	ND	ND	ND	ND
	Zn^2+^	ND	ND	9.8±0.4a	ND
	Cu^2+^	ND	ND	10.8±0.4a	ND
s2	Cd^2+^	22.3±1.6a	21.4±1.2a	ND	15.8±1.2a
	Zn^2+^	17.9±1.7a	ND	16.5±0.8b	13.2±1.0a
	Cu^2+^	ND	15.2±1.2a	17.3±1.0b	13.6±0.9a
s4	Cd^2+^	21.9±1.1a	3.9±0.1b	ND	9.1±0.6b
	Zn^2+^	16.9±1.0a	ND	15.7±0.5b	7.6±0.6b
	Cu^2+^	ND	2.8±0.0b	16.0±0.6b	7.5±0.5b
s5	Cd^2+^	6.3±0.4b	8.8±0.1c	ND	ND
	Zn^2+^	3.6±0.2b	ND	15.7±0.3b	ND
	Cu^2+^	ND	6.7±0.1c	16.2±0.4b	ND

^a^ Transgenic sugar beet lines overexpressing StGCS-GS (s2, s4 and s5) and wild-type sugar beets (wt) were treated with double or triple combinations of 50 μM CdCl_2_, ZnCl_2_ and CuCl_2_, designated as Cd-Zn, Cd-Cu, Zn-Cu and Cd-Zn-Cu. The roots of these plants were collected to analyze ion accumulation in triplicate. Values represent the means ± standard deviation with a 5% level of significance (*a-c*). ND: not detected.

## Discussion

Interest in the use of phytoremediation to ameliorate heavy metal pollution has increased due to its high efficiency and lack of secondary environmental pollution. Plants suitable for phytoremediation should have high rates of accumulation *in vivo*, fast growth, and high biomass. They should be simple to harvest and capable of accumulating a variety of ions. According to these criteria, the sugar beet (*Beta vulgaris* L.) is a nearly ideal choice for genetic engineering for bioremediation. In addition, sugar beet carbohydrates can be used as raw material for ethanol synthesis [17]. Thus, transgenic sugar beets have the potential for both heavy metal bioremediation and bioenergy production.

The low-molecular-weight molecule glutathione (GSH) is a ubiquitous non-protein thiol (NPT) with several pivotal roles in heavy metal ion homeostasis and detoxification. In numerous eukaryotes, GSH is synthesized via a sequential two-step pathway regulated by two peptide bond-forming enzymes: *γ*-GCS and GS. However, GSH synthesis is relatively uncommon among some bacteria. The first bifunctional *γ*-glutamylcysteine synthetase-glutathione synthetase (*γ*-GCS-GS) was identified in *Streptococcus agalactiae*, and its activity was shown to be insensitive to feedback inhibition by GSH [18]. *γ*-GCS-GS-like fusion proteins (GshF) in *Pasteurella multocida* and *Enterococcus italicus* also exhibited a high capacity for GSH production [19, 20]. Recently, in the *Streptococcus thermophilus* strain SDMCC18, inactivation of the gshF gene was found to result in significantly reduced growth and survival in the presence of reactive oxygen and H_2_O_2_; however, oxidative tolerance was restored by exogenous GSH [21]. In addition to the typical characteristic in GSH synthesis without limit inhibition by the product, the ectopic expression of StGCS-GS of *S*. *thermophilus* ATCC 19258 was confirmed to improve transgenic tobacco performance under oxidative and cadmium stresses due to a large increase in GSH content [14]. In addition, bacterial cells overexpressing StGCS-GS were found to have a high tolerance to and ability to accumulate Cd, Zn and Cu ions [15]. Based on these data, we constructed transgenic StGCS-GS-expressing sugar beet lines with the aim of evaluating their tolerance mechanism and potential for phytoremediation of various heavy metals.

After evaluation by semi-quantitative RT-PCR, we selected three lines (s2, s4 and s5) with high levels of StGCS-GS expression for metal tolerance studies (Fig 1). The transgenic plants did not exhibit any obvious phenotypic or growth differences under normal conditions compared to wt (Fig 2). However, the transgenic plants exhibited enhanced resistance to different concentrations of Cd, Zn, and Cu compared to wt, and their phenotypic differences became more pronounced as the metal ion concentrations increased, as indicated by changes in root length and fresh weight (Figs 3 and 4). The striking differences observed between the transgenic and control plants demonstrated the major contribution of StGCS-GS to heavy metal tolerance. However, the three transgenic lines exhibited different levels of tolerance to 50, 100 and 200 μM Cd, Zn and Cu. s2 was much more tolerant to these three heavy metals than s4 and s5 (Figs 2, 3 and 4), most likely because the expression level of StGCS-GS was highest in s2 (Fig 1). A similar result was observed in transgenic *GSH2*-overexpressing Indian mustard, in which Cd tolerance and accumulation were correlated with the level of expression of the target gene [8]. Among the three heavy metal ions, Cd was the most severely toxic to sugar beets, followed by Zn and Cu (Figs 2, 3 and 4). Cd is the most phytotoxic metal ion because of its high solubility, high level of absorption by plants, and radical introgression into the food chain, which causes serious human health hazards [22, 23]. Although both Zn and Cu are essential micronutrients, when these metals are present in excess, they can play a cytotoxic role by inducing oxidative stress, which leads to plant growth retardation [24].

To determine whether the transgenic sugar beets were capable of accumulating heavy metal ions and to identify the mechanism underlying the tolerance and accumulation patterns, we first examined the amount of Cd, Zn and Cu in both shoots and roots. Increased ion accumulation was observed in the upper parts of the transgenic plants, particularly in s2, which expressed the highest amount of StGCS-GS (Figs 1 and 5). The same accumulation pattern was observed in Indian mustard overexpressing *GSH1* and *GSH2* [8, 9]. These findings suggest that overexpression of StGCS-GS increases enrichment of Cd, Zn and Cu through transportation and accumulation of ions from roots to shoots. Thus, the high biomass production and the superior heavy metal tolerance and accumulation in transgenic sugar beets should enable the amelioration of heavy metal contamination when the plants are harvested.

Furthermore, the GSH levels were significantly higher in the StGCS-GS-transgenic lines than in wt under normal conditions, while the PC levels were almost unchanged (Fig 6). The increased activity of *γ*-GCS and GS enzymes induced by the overexpression of StGCS-GS may have led to a high level of GSH production under normal conditions [14]. Similarly, homologous overexpression of g-ECS in *Arabidopsis* resulted in approximately 2-fold GSH enrichment [25]. Upon exposure to heavy metal stresses, the GSH content decreased, and PC formation was activated to different degrees under different heavy metal stresses (Fig 6). GSH not only acts as an antioxidant to maintain the cellular redox state but also serves as a chelator of heavy metal ions to protect cellular molecules [26]. GSH detoxifies Cd by directly forming a GSH-Cd complex in Cd-treated yeast [27]. GSH is also a substrate for PC synthesis and is crucial for the detoxification of heavy metals such as Cd and Ni [28]. Thus, the high GSH levels achieved via overexpression of StGCS-GC with limited end-product feedback inhibition may enable increased accumulation of heavy metal ions in transgenic plants despite the toxic effects of Cd^2+^, Zn^2+^ and Cu^2+^, resulting in significantly improved tolerance to heavy metal toxicity in these transgenic sugar beets. These results are consistent with those of previous studies in which transgenic Indian mustard overexpressing *GSH1* and *GSH2* exhibited increased GSH and PC concentrations as well as increased Cd tolerance and accumulation compared to controls [8, 9]. This increased tolerance and accumulation were attributed to the enhanced PC production in the transgenic plants, which should enhance their capacity to detoxify and sequester heavy metal ions [28]. The differences in heavy metal tolerance and accumulation among the transgenic lines may be due to the different expression levels of StGCS-GS in these lines; these differences in expression result in different levels of GSH and PC production. We hypothesize that both the tolerance and accumulation of heavy metals would increase if GSH and PC contents were maintained in homeostasis when GSH production increases in transgenic plants.

In most heavy metal-polluted environments, several ions coexist with each other and jointly exert toxic effects on organisms. Therefore, we performed multiple heavy metal stress experiments employing Cd-Zn, Cd-Cu, Zn-Cu and Cd-Zn-Cu treatments. Compared to the single heavy metal experiments, combinations of two or three metals at a concentration of 50 μM caused severe damage to all tested lines. However, overexpression of StGCS-GS still improved the tolerance to and increased the accumulation of complex heavy metals (Fig 7; Tables 1 and 2). Cd, Zn and Cu were primarily concentrated in the shoots, without obvious competition among the metals (Tables 1 and 2). Cd, Zn and Cu are all divalent cations, and their structures and modes of complexation are similar; thus, there is no obvious antagonistic relationship among these ions. A similar phenomenon was observed in *Phaseolus vulgaris* plants, in which Cd-Zn exhibited a synergistic or additive effect that depended on the concentration of the ions in the medium [29].

Taken together, our results indicate that overexpression of StGCS-GS is an efficient means of increasing Cd, Zn and Cu accumulation in sugar beet shoots and enhancing heavy metal tolerance in transgenic plants without negatively affecting growth. Modification or overexpression of the enzymes involved in GSH synthesis is a good approach to enhance heavy metal tolerance and accumulation in plants. Taken together, the results of this study suggest that transgenic sugar beets overexpressing StGCS-GS seem to be excellent candidate plants for use in phytoremediation of heavy metal pollution.
